# Supplementary figures and images for: Gene length and detection bias in single cell RNA sequencing protocols
Source: F1000Res. 2017 Apr 28;6:595. [Version 1] doi: 10.12688/f1000research.11290.1 (PMC5428526; doi:10.12688/f1000research.11290.1)

# Unique genes: Average log-counts

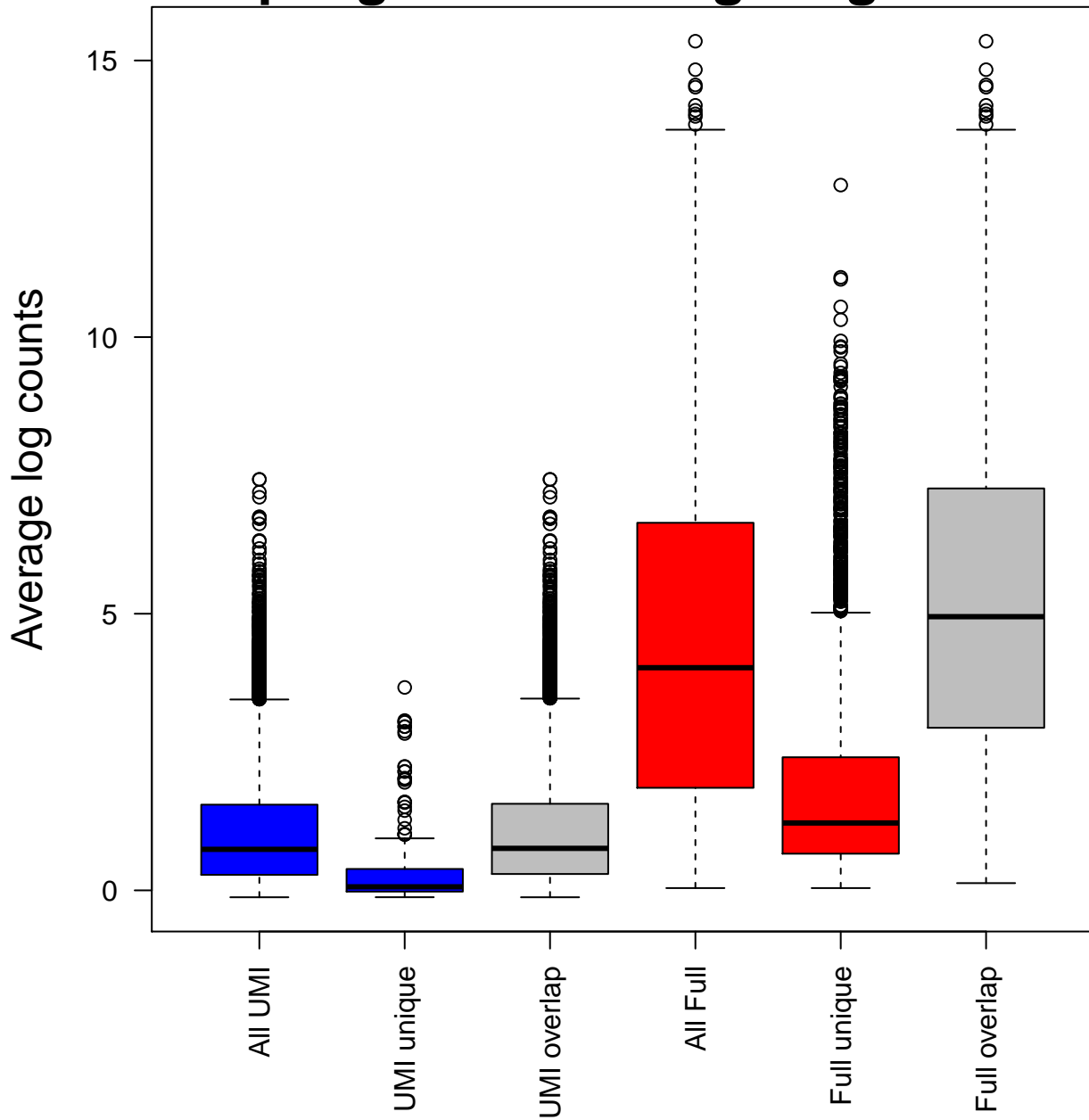

Supplement: Supplementary file 1 [file f1000research-6-12181-s0000.tgz › 5dbbedea-9028-4f4b-94b4-ef245529b6ed.pdf]
