# Supplementary material for: Gene length and detection bias in single cell RNA sequencing protocols
Source: F1000Res. 2017 Apr 28;6:595. [Version 1] doi: 10.12688/f1000research.11290.1 (PMC5428526; doi:10.12688/f1000research.11290.1)
Supplement: Peer review history

# Peer review: Referee response for version 1

**DOI:** 10.5256/f1000research.12181.r22437

**Published:** 2017-05-19

**Authors:** Sam Buckberry, Timothy Stuart

**Affiliations:** 1 Harry Perkins Institute of Medical Research, University of Western Australia, Perth, WA, Australia; 2 Australian Research Council Centre of Excellence in Plant Energy Biology, University of Western Australia, Perth, WA, Australia

Phipson, Zappia and Oshlack present evidence against the existence of systematic gene length bias in single cell RNA sequencing experiments that use unique molecular identifiers (UMI). In contrast, methods that measure read counts across full length transcripts appear similar to bulk RNA sequencing methods in that they are biased against short transcripts. Although these results are somewhat unexpected by those working in the field, the thorough analysis presented by Phipson et al. will be a valuable reference to those wishing to design single cell RNA-seq experiments.  The article is written in a clear and accessible manner, and it is also nice to see all the analysis code has been made available. However, there are a number of minor issues with the paper in it's current form that we think should be addressed.

 Of particular note, the paper appears to conflate UMI methods with 3' counting methods. We see this as incorrect as i) long-read sequencing technology may allow profiling of full-length transcripts while incorporating UMIs, and ii) 3' counting methods can be used without UMIs. The effect of 3' counting on gene length bias could be separated from the effect of using UMIs by ignoring UMIs in a 3' counting experiment and testing to see if substantial gene length bias exists. Our guess is that it would not, due to the simple fact that the effective gene length is approximately equal for all genes when you measure only the last ~300 bp. Therefore, for the examination of gene length bias, it seems to us that the emphasis should be on 3' counting and not UMIs. Of course, not using UMIs would introduce substantial PCR amplification bias, but this is a separate issue to that being addressed by the paper.

 Minor comments:

 Introduction:

 "...technology enables researchers to examine transcription at the resolution of a single cell..." -- The technology measures mRNA abundance, not transcription itself. (paragraph 1)

 "...alternative splicing... analysis is not possible with data generated with protocols that include UMIs." -- it is possible that long-read technologies (eg. Pacbio or Oxford Nanopore) could be coupled with UMI tagged cDNA generated using drop-seq methods before cDNA fragmentation to capture full-length transcripts. (paragraph 3)

 It may be beneficial to include supplemental table 1 in the main text.

 Processing of all datasets:

 Why are different cutoffs used for filtering out cells between experiments? eg. 80% dropout for Kolodziejczyk, 85% dropout for Guo, 90% for Camp, 70% for Tung, 85% Klein. Similar with the library size cutoff and percent ERCC cutoff.

 In the Klein methods section, ERCC percentage is reported as >0.01 total library size rather than the percentage. For readability it may be better to have consistent style throughout the manuscript (eg. percent total for everything).

 For Ziegenhain methods, it's stated that all cells appeared high quality and so weren't filtered. What constitutes high quality, and how was this assessed? As the count matrix was used in this case, were the cells pre-filtered by the original authors?

 Statistical analysis:

 "UMI dataset was normalized using scran ...as it clearly showed composition bias." What method was used for normalization, and what exactly is meant by 'compositional bias' and how was this assessed? We believe the scran package depends on scater for implementation of it's normalization methods.

 Why use different fold change parameters for UMI and full-length methods? Also, a log (is this log2?) fold change of 1 is 0 fold change. Furthermore, how were the log transformed values calculated for datasets with many zeros?

 Figure 1:

 More informative axis labels, eg. "Average normalized read counts (log2 scale)" rather than "AvgLogCounts" would increase readability. 

 Please note that the Tung et al. paper is now published in Scientific Reports and the Ziegenhain paper is published in Molecular Cell.

 Figure 4:

 The comparison of the number of genes detected by UMI vs full-length methods is somewhat confounded by the differing sequencing depth between the methods. This is stated in the text, but a better comparison could be made by sub-sampling reads from the experiments to equivalent numbers of reads per cell.

We have read this submission. We believe that we have an appropriate level of expertise to confirm that it is of an acceptable scientific standard.

# Peer review: Referee response for version 1

**DOI:** 10.5256/f1000research.12181.r22371

**Published:** 2017-05-17

**Authors:** Wolfgang Huber

**Affiliations:** 1 EMBL Genome Biology Unit, European Molecular Biology Laboratory, Heidelberg, Germany

The paper presents a very useful investigation of the dependence of detection efficiency in single cell RNA sequencing on (a) gene length and (b) certain choices in the experimental protocol, namely, shotgun sequencing of full transcripts versus transcript end sequencing such as in methods that employ UMIs. Overall the article is well written, clear, and likely to be useful for practitioners of experimental design and data analysis in the field.

 I have a few points that a revised version might address:

  

1. The term ‘dropout’ is used in many places, but not properly defined, neither mathematically nor biophysically. At some point in the middle of the manuscript the authors seem to imply that they use ‘dropout’ as a synonym to ‘occurrence of a zero count’ in the data. What is the rationale behind giving the name ‘dropout’ to such an event? What is dropping, and out of what? I understand that some colleagues use this term to point to high probabilities of seeing a zero count for (low abundant?) genes due to the sparse sampling, but I wonder whether (or in which datasets, protocols) this is really something that is more ominous than what is trivially implied by Poisson or Gamma-Poisson statistics, and if so, whether only 0s are ominous or also 1s, 2s, …? Given that this is a paper by statisticians on detection biases it would be great to see a more careful treatment of this aspect of the data.
2. Why are the parameter choices in Section “Processing of all datasets” (for fraction of dropouts and number of reads) so different between the different datasets? There seems to be a potential for the introduction of biases or artefacts in the computed statistics (of Figs. 1 and 2) through choices made here, and it would be good to demonstrate that such biases, if any, are inconsequential.
3. In Figs. 1 and 2, how are the ‘average log counts’ computed for data that contain a lot of zeros? The logarithm is not defined for 0. And whatever is the answer to this question, how did the authors make sure that it introduces no biases/artefacts that affect the shown trends? In particular, in conjunction with the filtering steps mentioned above in Point 2?
4. In Figs. 1 and 2, how is the set of genes selected that enter the calculation of ‘Proportion of zeros in each gene’? Again, how can we be sure that the choices made in the filtering do not affect the conclusions made here?
5. It is recommendable that the scripts are provided in a github repository. I wonder whether the authors would be willing to go the full length and upload the scripts to a repository that also does regular “live” testing of the scripts for functionality (e.g. installation, dependencies, versions, data availability), such as Bioconductor or CRAN.
6. On p.5., the authors report differing trends for human PGCs and human brain organoids, compared to mouse ESCs. Do they imply that this is a biological observation, and if so, what does it mean? Or could there be confounding with experimental circumstances? (In which case the effect would perhaps better be reported in association with that than with the names of biological conditions).
7. In the Discussion and on p.5, results from applying RPKM to UMI-based data are reported. Perhaps the point could be strengthened that already for very basic theoretical reasons this is a nonsensical thing to do. Finding this also empirically is nice, but perhaps it can be said that this confirms basic reasoning rather than being ‘news’.

 

**Minor:**

 On p.1, a wording is used that implies that datasets are being sequenced. But nucleotides are sequenced, and datasets are produced.

 I think the term “pseudo-aligned / pseudo-alignment” is ugly, and “mapped / mapping” is better and more widely used in the field.

 On the bottom right of p.4, the term “log-fold change cut-off of 1” is unclear. Which base? Also, do you perhaps mean *absolute* logarithmic fold change?

 The boxplots in Figs. 1 and 2 are a bit dull. Use of geom_hex with aes(x=rank(genelength)) in ggplot2 could present an alternative.

 In the caption of Fig.1, ambiguity in the term ‘log counts corrected by gene length’ could be avoided by more explicit mathematical terminology (e.g. corrected = divided?)

 Discussion: the conclusion that the choice of protocol may affect which pathways can be studied is a bit wild, and probably also not helpful if not translated into concrete advice to readers for how to address it when doing their experimental designs.

I have read this submission. I believe that I have an appropriate level of expertise to confirm that it is of an acceptable scientific standard.

# Peer review: Referee response for version 1

**DOI:** 10.5256/f1000research.12181.r22438

**Published:** 2017-05-10

**Authors:** Samuel W. Lukowski

**Affiliations:** 1 Institute for Molecular Bioscience (IMB), The University of Queensland, St Lucia, Qld, Australia

**General comments**

 This is a well-written and concise study that reveals some very interesting results. Firstly, pertaining to the enrichment of biological processes in data processed using different protocols, the fact that the genes detected in full-length transcript and 3' transcript-end protocols show markedly different specificity for enriched pathways is intriguing. With 3' being highly specific and biologically relevant, compared to the generic pathways identified for full-length data, it raises the possibility that a much higher resolution of biological function and greater classification accuracy might be attained if full-length transcript data was re-analyzed as 3' transcript-end. Secondly, the importance of using correct normalization methods for UMI data is shown here to be of critical importance for accurate analysis.

 Also, many thanks to the authors for making their analysis code available on GitHub.

**Summary**

 In this manuscript, the authors asked whether single-cell RNA-seq data would be biased by choice of protocol. Specifically, they looked at the difference between data generated using full-length transcript protocols compared to those generated using 3' transcript end-only protocols that incorporate unique molecular identifiers. To do this, they used publically available scRNA-seq datasets from mouse and human.

 Their conclusion is that full-length transcript methods exhibit gene length bias, such that short genes have less mapped reads than longer genes, which translates to lower transcript counts and a higher dropout rate. Conversely, UMI-based methods do not suffer from either of these effects. They also demonstrated that a combination of both methods can enhance the biological interpretation of the scRNA-seq data.

**Comments for the authors:**

1. For each of the datasets that were pre-processed (not raw data), it is possible that the different reference genomes (hg19, complete GRCh38, transcriptome-only GRCh38) and the use of different software packages could create artifacts that affect data analysis, particularly if the mapping software was an old version. I note that, with respect to pre-processed data, five different aligners were used. It is clear that all alignment packages have their strengths/weaknesses, especially if they haven't been updated regularly. Could the differences between (i) these packages, and (ii) the different references, contribute in any way to the results obtained in this study?
2. Related to question 1, would isoform/ splice junction-aware aligner yield different results compared to those that aren't designed for that type of mapping? Would you expect a difference in the full-length data sets that were processed with transcriptome-only reference (Guo 2015 ^1^) compared to the complete hg38 genome reference (Camp 2015 ^2^)?
3. Each pre-processed dataset was filtered using slightly different parameters. How did the authors establish the dropout percentage threshold for removing cells (none, 70, 80, 85)? How were the library size and sequencing read thresholds determined for each sample? It's not clear to me why these should all be different. Is it to maximize the cell numbers on a per-sample basis? Have the authors tried using the same threshold for all pre-processed samples as for the in-house filtering (90%), or applying the other thresholds to raw data?
4. Gene ontology analysis is widely used and can provide insight into biological functions. I wonder whether the authors also considered using more specific databases such as Reactome or KEGG that can highlight enriched pathways that are not detected by GO analysis. These may show less disparity than GO terms. 
5. Minor point: Throughout the text and in Figure 3 and 4, UMI is capitalised, but in Fig 2 it is shown in lower case in the plot titles.

I have read this submission. I believe that I have an appropriate level of expertise to confirm that it is of an acceptable scientific standard.

## References

[1] GuoFYanLGuoHLiLHuBZhaoYYongJHuYWangXWeiYWangWLiRYanJZhiXZhangYJinHZhangWHouYZhuPLiJZhangLLiuSRenYZhuXWenLGaoYQTangFQiaoJ: The Transcriptome and DNA Methylome Landscapes of Human Primordial Germ Cells. *Cell*.2015;161(6) : 10.1016/j.cell.2015.05.015 1437-52 10.1016/j.cell.2015.05.015 26046443

[2] CampJGBadshaFFlorioMKantonSGerberTWilsch-BräuningerMLewitusESykesAHeversWLancasterMKnoblichJALachmannRPääboSHuttnerWBTreutleinB: Human cerebral organoids recapitulate gene expression programs of fetal neocortex development. *Proc Natl Acad Sci U S A*.2015;112(51) : 10.1073/pnas.1520760112 15672-7 10.1073/pnas.1520760112 26644564PMC4697386

# Peer review: Referee response for version 1

**DOI:** 10.5256/f1000research.12181.r22376

**Published:** 2017-05-10

**Authors:** Charlotte Soneson

**Affiliations:** 1 Institute of Molecular Life Sciences, University of Zurich (UZH), Zürich, Switzerland

This is a nice evaluation of the extent of gene length and detection bias in single-cell RNA-seq data sets generated with different types of protocols. Overall, it is clearly written and the results are well presented and agree with expectations. All analysis code is available in a GitHub repository. An additional step towards full reproducibility would be to also make the processed data objects and additional scripts, not present in the GitHub repository, accessible. 

 Otherwise, my main comment concerns the representation of gene abundances, specifically the calculation of RPKMs by dividing the library size-normalized gene counts with the "exon-union" length of the gene. Without information about which isoform is contributing to the expression of a given gene, this length may be far from the true number of base pairs "contributing" to the observed reads. An alternative approach would be to aggregate isoform-level TPM estimates (from methods like Salmon ^1^, RSEM ^2^ or kallisto ^3^) to the gene level, and I am wondering whether that would affect the conclusions. Similarly, it could be interesting to investigate whether suggested alternatives to actual or expected read counts, such as "scaled TPMs" ^4^ or census counts ^5^, would mitigate the observed gene length bias. 

 In a couple of places, I think that the manuscript would benefit from some clarifications:

- In the last lines of the "Gene filtering" paragraph, it is mentioned that genes that could not be annotated with gene length information were filtered out. How many genes are affected by this, and in what way can they be assigned reads (i.e., correspond to well-defined genomic regions) but not a length? 
- From the "Processing of all datasets" paragraph, it is not completely clear whether cells are filtered out only if they have both more than (e.g.) 85% dropout and fewer than (e.g.) 500,000 reads, or if one of these criteria alone is enough. It is also not fully clear from the text whether cell filtering or gene filtering was performed first (e.g., the "Gene filtering" paragraph mentions "all cells", but in the following paragraph and in the code it seems that the cell filtering was performed first).
- On what values was the principal component analysis applied? Could you expand a bit more on how the data set merging strategy ensures that the larger datasets do not dominate the PCA (they still make up a larger part of the final dataset)? 
- In the "Statistical analysis" paragraph, how was the UMI data set normalized with scran? Was there an actual normalization step, or a calculation of normalization factors used later in the analysis? 
- For the four mouse mESC data sets, it might be useful to provide a table listing the conditions (=colors in Figure 3b) that were included in each of them, since it is a bit difficult to discern all color/symbol combinations in Figure 3b.
- The numbers in Figure 4a and b do not match (the numbers in Figure 4b match those given in the text, while those in Figure 4a match the figure legend). 
- Are the two densities in Figure 4d generated with the same kernel width? If not, the differences may be visually exaggerated. 
- For the preprocessing of the Guo et al. data set, the pseudo-alignment with Salmon was done to the reference transcriptome rather than the genome.
- Finally, for the gene set analysis, in addition to the observation that there are some gene sets with short median gene lengths that are among the most enriched in the "UMI-specific" genes, it might be interesting to see whether these gene sets were in fact top-ranked because of the short genes contained in them, or if it was the longer genes in these gene sets that were the significant ones.

I have read this submission. I believe that I have an appropriate level of expertise to confirm that it is of an acceptable scientific standard.

## References

[1] PatroRDuggalGLoveMIIrizarryRAKingsfordC: Salmon provides fast and bias-aware quantification of transcript expression. *Nat Methods*.2017;14(4) : 10.1038/nmeth.4197 417-419 10.1038/nmeth.4197 28263959PMC5600148

[2] LiBDeweyCN: RSEM: accurate transcript quantification from RNA-Seq data with or without a reference genome. *BMC Bioinformatics*.2011;12: 10.1186/1471-2105-12-323 323 10.1186/1471-2105-12-323 21816040PMC3163565

[3] BrayNLPimentelHMelstedPPachterL: Near-optimal probabilistic RNA-seq quantification. *Nat Biotechnol*.2016;34(5) : 10.1038/nbt.3519 525-7 10.1038/nbt.3519 27043002

[4] SonesonCLoveMIRobinsonMD: Differential analyses for RNA-seq: transcript-level estimates improve gene-level inferences. *F1000Res*.2015;4: 10.12688/f1000research.7563.2 1521 10.12688/f1000research.7563.2 26925227PMC4712774

[5] QiuXHillAPackerJLinDMaYATrapnellC: Single-cell mRNA quantification and differential analysis with Census. *Nat Methods*.2017;14(3) : 10.1038/nmeth.4150 309-315 10.1038/nmeth.4150 28114287PMC5330805
